# Epidemiological characteristics and influencing factors of tuberculosis aggregation in schools in Wuhan, China during 2017–2022

**DOI:** 10.3389/fpubh.2024.1365983

**Published:** 2024-06-04

**Authors:** Zhengbin Zhang, Gang Wu, Aiping Yu, Jing Hu, Wei Zhang, Zhouqin Lu, Jun Wu, Lina Wang, Xiaojun Wang, Jianjie Wang, Guiyang Wang, Yuehua Li, Meilan Zhou

**Affiliations:** ^1^Department of Tuberculosis Control, Wuhan Pulmonary Hospital, Wuhan, Hubei, China; ^2^Infectious Disease Prevention and Control Department, Dongxihu Centers for Disease Prevention and Control, Wuhan, Hubei, China; ^3^Center for Disease Control and Prevention of Yangtze River Navigation Administration, Wuhan, Hubei, China; ^4^School of Computer and Engineering, Communication Zhengzhou University of Light Industry, Zhengzhou, Henan, China

**Keywords:** pulmonary tuberculosis, school, outbreak, epidemiology, regression analysis

## Abstract

**Background:**

Wuhan is located in the hinterland of China, in the east of Hubei Province, at the intersection of the Yangtze River and Hanshui River. It is a national historical and cultural city, an important industrial, scientific, and educational base, and a key transportation hub. There are many schools in Wuhan, with nearly a thousand of all kinds. The number of students is ~2.2 million, accounting for nearly one-fifth of the resident population; college or university students account for ~60% of the total student population. The geographical location of these colleges is relatively concentrated, and the population density is relatively high, making it prone to tuberculosis cluster epidemic.

**Objective:**

This study analyzed the epidemiological characteristics and influencing factors of tuberculosis aggregation in schools in Wuhan, China, during 2017–2022 to provide the basis for the scientific development of tuberculosis prevention and control strategies and measures in schools.

**Methods:**

This study adopted the methods of descriptive epidemiology to analyze the epidemic characteristics of tuberculosis aggregation in schools in Wuhan from January 2017 to December 2022, collecting the relevant data on tuberculosis prevention and control in all kinds of schools in the city using Questionnaire Star, an application of the China network questionnaire survey, and analyze the influencing factors of tuberculosis aggregation by using multifactor logistic regression analysis.

**Results:**

From 2017 to 2022, 54 outbreaks of pulmonary tuberculosis aggregation in schools were reported in Wuhan, which involved 37 different schools, including 32 colleges or universities and five senior high schools; 176 cases were reported, among which 73 were positive for pathogens and 18 were rifampicin or izoniazid resistant. The median duration of a single cluster epidemic was 46 (26,368) days. Universities were more prone to cluster outbreaks than middle schools (**χ^2^** = 105.160, *P* = 0.001), and the incidence rate among male students was higher than that of female students in cluster epidemics (**χ^2^** = 12.970, *P* = 0.001). The multivariate logistic regression analysis results showed that boarding in school (OR = 7.60) is the risk factor for a tuberculosis cluster epidemic in schools. The small number of students (OR = 0.50), the location of the school in the city (OR = 0.60), carry out physical examinations for freshmen (OR = 0.44), carry out illness absence and cause tracking (OR = 0.05), dormitories and classrooms are regularly ventilated with open windows (OR = 0.16), strict implement the management of sick student's suspension from school (OR = 0.36), and seeking timely medical consultation (OR = 0.32) were the protective factors for a tuberculosis cluster epidemic in schools.

**Conclusion:**

We successfully identified the epidemiological characteristics and influencing factors of tuberculosis aggregation in schools in Wuhan. The results revealed the influence and status of various factors and indicated ways for schools to improve their TB prevention and control measures in their daily activities. These measures can effectively help curb the cluster epidemic of tuberculosis in schools.

## Introduction

Tuberculosis is a chronic infectious disease caused by mycobacterium tuberculosis. It is one of the three major infectious diseases in China. A school is a place where people are relatively concentrated. When any student or teacher is infected with tuberculosis and if it is not discovered in time, it may result in a tuberculosis epidemic (1–3), disrupting the normal teaching schedule of the school. If it is not handled properly, it will even cause negative public opinion, resulting in adverse social impact.

Wuhan is located in the east of Hubei Province, at the intersection of the Yangtze and Hanshui Rivers. The maximum horizontal distance between east and west is 134 km, and the maximum vertical distance between north and south is nearly 155 km. As the economic and geographical center of China, Wuhan is known as the “thoroughfare of nine provinces.” Its high-speed rail network radiates over half of China, making it the only city in Central China to have direct flights to five continents. There are several schools in Wuhan, with nearly a thousand of all kinds, and the number of students of all kinds is ~2.2 million, accounting for nearly one-fifth of the resident population. College students account for approximately 60% of the total student population (4). Due to the large number of schools, concentrated geographical location, dense student population, and high mobility in Wuhan, nearly 70% of the students come from the surrounding cities of Wuhan, some of which are areas with high tuberculosis epidemics. Given that the understanding of tuberculosis prevention and control in various schools is uneven, there are frequent clustered outbreaks of tuberculosis in schools, making the task of tuberculosis prevention and control by disease control departments extremely arduous. This study, therefore, aims to analyze the epidemiological characteristics and influencing factors of a tuberculosis cluster epidemic in Wuhan from 2017 to 2022 and provide a basis for the scientific formulation of strategies and measures to prevent and control tuberculosis in schools.

## Methods

### Data collection

We retrospectively collected the data of clustered epidemic cases of tuberculosis in schools investigated and disposed of by the centers for disease control and prevention in Wuhan from 2017 to 2022. The data included case questionnaires, close contact screening forms, and epidemiological investigation reports involving 37 schools relating to a total of 54 clustered epidemics. A self-made school tuberculosis prevention and control checklist was prepared, referring to the School Tuberculosis Prevention and Control Checklist. Based on these data, combined with the purpose of this study, the final questionnaire was prepared. The survey was conducted through the “Questionnaire Star” network platform. The questionnaire was distributed by the local school prevention and control QQ group of the centers for disease control and prevention (tuberculosis prevention and control) on WeChat and included questions on the school name, address, nature, type, number of students, whether the prevention and control system was established, and whether the epidemic reporter was clear and the school was configured.

### Data analysis index

A retrospective cohort study was used to analyze the data of the school tuberculosis epidemics in Wuhan from 2017 to 2022. It focused on analyzing the incidence trend, time distribution, regional distribution, gender and age distribution, clinical symptoms, laboratory examination, and the factors influencing cluster epidemics.

### Quality control

The questionnaire was finalized after full discussion, repeated argumentation, and revision by TB prevention and control experts. The disease control and prevention centers in all districts (tuberculosis control centers) received unified training and were made familiar with the survey plan and contents. The training emphasized matters needing attention through the QQ group of tuberculosis in district schools, the need to adopt a unified guiding language, answer the questions of school doctors or teachers, and the need to avoid suggestive language. After the school doctors or teachers filled out the questionnaire, they were to submit it on time after checking it for accuracy or correcting any problems promptly. This process was suggested to ensure the quality of the questionnaires.

### Related definition

#### Definition of the clinical symptoms of pulmonary tuberculosis

According to the People's Republic of China (PRC) Health Industry Standard-Tuberculosis Classification (WS 196–2017) (5), clinical symptoms are defined as cough and expectoration for more than 2 weeks, hemoptysis, chest tightness, chest pain, fever, night sweats, fatigue, and weight loss, among others.

#### Definition of pulmonary tuberculosis case diagnosis

All cases were based on the standard of the People's Republic of China (PRC) Health Industry Standard-Tuberculosis Diagnosis (WS 288-2017) (5). Pulmonary tuberculosis refers to the tuberculous lesions in the lung tissue, trachea, bronchus, and pleura. The diagnosis was made by a tuberculosis diagnosis team composed of two senior clinicians, one radiologist, one laboratory technician, and one epidemiological public health doctor. Based on pathogen (including bacteriology and molecular biology) examination, along with epidemiological history, clinical manifestations, chest imaging, relevant auxiliary examinations, and differential diagnosis, a comprehensive analysis was conducted to make a diagnosis.

#### Definition of latent infection of Mycobacterium tuberculosis

The human body is infected with *Mycobacterium tuberculosis*, but there are no clinical symptoms or signs of tuberculosis. There is no evidence of active tuberculosis in clinical bacteriology and imaging. Although the vast majority of people infected with *Mycobacterium tuberculosis* do not have the symptoms and signs of tuberculosis and are not infectious, they are at risk of progressing to active tuberculosis.

#### Definition of tuberculosis cluster epidemic

According to the “Guidelines for the Prevention and Control of Tuberculosis in Schools in China (2020 Edition),” when three or more cases with epidemiological correlation are found in the same school in the same semester, the county-level disease prevention institutions should report it to the superior health administrative departments, superior disease prevention and control institutions, and schools and write epidemic investigation reports that should include data on the investigation and disposal of tuberculosis epidemic in schools. We conventionally define it as a cluster epidemic (6).

#### Definition of medical treatment delay for sick students

According to the literature reviews (7–9), it is defined as a medical treatment delay when students have not seen a doctor in a medical and health institution for more than 9 days after they have experienced symptoms.

#### Definition of window ventilation reaching the standard

Bedrooms, classrooms, and other public places are ventilated twice or more every morning and afternoon for at least 60 min each time.

### Data analysis

A descriptive epidemiological method was used to analyze the situation of the school tuberculosis epidemic in Wuhan. Questionnaires and Excel 2016 were used to build and input the data. SPSS25.0 was used to analyze the data by **χ^2^** test, Trend chi-square test or precise probability method, and multivariate unconditional logistic regression. The ratio (OR) and 95% confidence interval (CI) were used to estimate the connection strength between each factor and the clustering epidemic situation, and the test level was α = 0.05.

## Results

### Epidemic characteristics of pulmonary tuberculosis in schools during 2017–2022

From 2017 to 2022, a total of 1,830 registered students with active pulmonary tuberculosis were found in Wuhan, among which 816 cases were pathogen-positive, accounting for 5.43% (1,830/33,682) and 4.62% (816/17,668) of the total population with active pulmonary tuberculosis. The registered number of students with active pulmonary tuberculosis gradually decreased from 20.75/100,000 in 2017 to 9.91/100,000 in 2022. However, the registered rate of pathogen-positive discovery gradually increased from 4.23/100,000 to 7.91/100,000 in 2020 and then declined to 5.52/100,000 in 2022. There were 54 cluster outbreaks, and 278 student cases were identified, accounting for 15.19% (278/1,830) of the total students (Table 1).

**Table 1:** Epidemic situation of pulmonary tuberculosis in schools in Wuhan from 2017 to 2022.

**Year**	**Number of registered cases of pulmonary tuberculosis in the whole population**		**Registration rate of pulmonary tuberculosis in the whole population (1/100,000)**		**Number of registered cases of pulmonary tuberculosis among students**		**Registration rate of pulmonary tuberculosis among students (1/100,000)**		**Student Clustering epidemic**	
	**Active pulmonary tuberculosis**	**Pathogen positive**	**Active pulmonary tuberculosis**	**Pathogen positive**	**Active pulmonary tuberculosis**	**Pathogen positive**	**Active pulmonary tuberculosis**	**Pathogen positive**	**Number of outbreaks**	**Number of pulmonary tuberculosis cases**
2017	6,074	2,216	55.76	20.34	397	81	20.75	4.23	5	31
2018	5,952	3,082	53.71	27.81	314	141	15.87	7.13	12	45
2019	5,937	3,172	52.95	28.29	320	148	15.43	7.14	16	81
2020	4,922	2,684	39.93	21.56	300	173	13.72	7.91	4	19
2021	5,597	3,315	41.01	24.29	271	146	11.78	6.35	11	60
2022	5,200	3,199	37.85	23.28	228	127	9.91	5.52	6	42
Total	33,682	17,668	46.13	24.2	1,830	816	14.35	6.40	54	278

### Characteristics of student cases with pulmonary tuberculosis cluster epidemic

The survey involved 994 schools in Wuhan, including 574 in urban areas (Jiang ‘an District, Jianghan District, Qiaokou District, Hanyang District, Wuchang District, Qingshan District, Hongshan District, and Donghu High-tech Zone) and 420 in suburb areas (Hannan District, Dongxihu District, Caidian district, Jiangxia District, Huangpi district, and Xinzhou District). From 2017 to 2022, a total of 54 cluster outbreaks of tuberculosis in school in Wuhan were reported, involving 37 different schools, including 24 outbreaks in Hongshan District (44.44%), six in Donghu High-tech Zone (11.11%), three each in Wuchang District (5.56%) and Qiaokou District (5.56%), and two outbreaks in Hanyang District (3.57%), involving 27 schools. There were five outbreaks in Dongxihu District (9.26%), three each in Xinzhou District (5.56%), Jiangxia District (5.56%) and Huangpi District (5.56%), and two outbreaks in Hannan District (3.70%), involving 10 schools. There were at least 3–21 cases in a single cluster epidemic among students aged 15–26 years old. The average epidemic duration was 35.42 ± 12.53 days, with a median of 46 (26,368) days.

### Regional distribution of cluster epidemic situation of pulmonary tuberculosis

From 2017 to 2022, there were 38 cluster epidemic cases of tuberculosis in the near urban area, involving 24 schools (24/574). There were 16 outbreaks in the far city involving 10 schools (10/420), with no statistical significance between them (**χ^2^** = 2.379, *p* = 0.123). There were 196 cases of students with cluster epidemic in the near city and 82 cases in the far city, and there was an obvious statistical significance in the change in the trend of the number of cases found in the 6 years (*Z* = 12.170, *p* = 0.033).

### Gender distribution of students with pulmonary tuberculosis cluster epidemic

From 2017 to 2022, there were 203 male cases and 75 female cases in the cluster epidemic of tuberculosis in Wuhan, and 3,124 male and 1,844 female close contacts involved in the tuberculosis epidemic were screened; the incidence rate of a tuberculosis cluster epidemic among male students was significantly higher than that of female students in the school (**χ^2^** = 12.970, *p* = 0.000). However, there was no statistical significance in the change in the trend of the number of male and female cases during the 6 years (*Z* = 7.665, *p* = 0.176) (Table 2).

**Table 2:** Analysis of the characteristics of tuberculosis cluster epidemic among students in Wuhan from 2017 to 2022.

**Year**	**Number of students with pulmonary tuberculosis**	**Region**		**Gender**		**Learning phase**		**Case discovery mode**		
		**Urban district**	**Suburb district**	**Male**	**Female**	**University**	**Middle/Senior school**	**Health examination**	**Seek medical attention for symptoms**	**Close contact screening**
2017	31	18 (58.06)	13 (41.94)	27 (87.10)	4 (12.90)	27 (87.10)	4 (12.90)	6 (19.35)	10 (32.26)	15 (48.39)
2018	45	35 (77.78)	10 (22.22)	35 (77.78)	10 (22.22)	41 (91.11)	4 (8.89)	12 (26.67)	13 (28.89)	20 (44.44)
2019	81	54 (66.67)	27 (33.33)	60 (74.07)	21 (25.93)	71 (87.65)	10 (13.35)	15 (18.52)	21 (25.93)	45 (55.56)
2020	19	9 (47.37)	10 (52.63)	14 (73.68)	5 (26.32)	10 (52.63)	9 (47.37)	2 (10.53)	6 (31.58)	11 (57.89)
2021	60	48 (80.00)	12 (20.00)	37 (61.67)	23 (38.33)	43 (71.67)	17 (28.33)	10 (16.67)	14 (23.33)	36 (60.00)
2022	42	32 (76.19)	10 (23.81)	30 (71.43)	12 (28.57)	37 (88.10)	5 (11.90)	8 (19.05)	9 (21.43)	25 (59.52)
Total	278	196 (70.50)	82 (29.50)	203 (73.02)	75 (26.98)	229 (82.37)	49 (17.63)	53 (19.06)	73 (26.26)	152 (54.68)
*Z*-value		12.170		7.665		21.658		5.231		
*p*-value		0.033		0.176		0.001		0.875		

Figures in brackets are composition ratio (%).

### Distribution of student stages in school with cluster outbreaks of tuberculosis

From 2017 to 2022, 32 universities, five middle schools, and 0 primary schools in Wuhan reported tuberculosis cases. The proportion of universities experiencing outbreaks of three or more student tuberculosis cases was 32.65% (32/98), and that in middle schools was 1.36% (5/369). There was a significant difference between them (**χ^2^** = 105.160, *p* = 0.000). There was no cluster epidemic in the 527 primary schools. According to the school segment, the incidence rate of tuberculosis in universities was significantly higher than that in middle schools, with statistical differences. There were 229 cases of students with cluster epidemic in universities and 49 cases in middle schools. The trend of the number of cases found in the 6 years was statistically significant (*Z* =21.658, *p* = 0.001) (Table 2).

### Methods of finding cluster epidemic cases of tuberculosis

From 2017 to 2022, 154 cases were found among students with tuberculosis cluster epidemic by close contact screening in Wuhan, accounting for 54.68% (157/278), followed by 73 cases with symptoms, accounting for 26.26% (73/278), and 53 cases with health examination, accounting for 19.06% (53/278). There was no statistical significance in the change in trend of the number of cases found in the 6 years (*Z* = 5.231, *p* = 0.875) (Table 2).

### Clinical symptoms and pathogenic characteristics of pulmonary tuberculosis aggregation cases

Among the 278 confirmed patients, there were 102 cases with pulmonary tuberculosis symptoms, and the incidence of symptoms was 36.69%. Among them, 62 cases were pathogen-positive, and 40 were pathogen-negative; in the asymptomatic cases of pulmonary tuberculosis, 11 were pathogen-positive and 165 were pathogen-negative. The incidence of pathogen-positive symptoms was higher than pathogen-negative, with statistical significance (**χ^2^** = 99.179, *p* = 0.001). The pathogen was negative in 205 cases and positive in 73 cases, among which 17 cases were resistant to rifampicin, one was resistant to isoniazide, and the resistance rate of rifampicin or isoniazide was 6.47%(18/278). The resistance rates of rifampicin among students in high schools and universities were 6.12% (3/49) and 6.55% (15/229), respectively. However, there was no statistical difference between the resistance rates of rifampicin among students in high schools and universities (**χ^2^** = 0.001, *p* = 1.000).

### Analysis of influencing factors of cluster epidemic of pulmonary tuberculosis in schools

There are 994 schools at all levels in Wuhan, and all schools were included in the survey scope through administrative intervention from the education bureaus of various districts in Wuhan. The previous occurrence of a tuberculosis cluster epidemic in schools was the dependent variable (1 = yes, 0 = no). School-related factors were independent variables (school nature: 1 = public; 0 = private; school accommodation: 1 = boarding; 0 = non-boarding; the number of students in school: < 600 students = 1, ≥600 students = 0; school infirmary: 1 = Yes, 0 = No; school doctors: 1 = Yes, 0 = No; school location: 1 = urban area, 0 = rural area; establish an infectious disease prevention and control system: 1 = Yes, 0 = No; carry out physical examination for freshmen: 1 = Yes, 0 = No; carry out illness absence and cause tracking registration: 1 = Yes, 0 = No; carry out TB prevention and control knowledge propaganda: 1 = Yes, 0 = No; dormitories and classrooms are regularly ventilated with open windows: 1 = Yes, 0 = No; and sick students are suspended from school: 1 = Yes, 0 = No). Multivariate logistic regression analysis was conducted with univariate analysis of statistically significant variables (school boarding, number of students, school location, carry out physical examination for freshmen, carry out illness absence and cause tracking registration, whether the students seek timely medical consultation, whether the dormitories and classrooms are regularly ventilated with open windows, and strictly implement the management of sick student's suspension from school) as independent variables. The results revealed that all the significant variables were influencing factors of tuberculosis aggregation in schools (Tables 3, 4).

**Table 3:** Univariate logistic regression analysis of tuberculosis aggregation in schools in Wuhan (*n* = 994).

**Independent variable**	**β-value**	**Standard deviation**	**Wald *χ^2^***	***p*-value**	**OR (95% CI)**
School nature	0.365	0.209	3.041	0.081	1.44 (0.96–2.17)
School boarding	2.067	0.213	94.016	0.001	7.90 (5.20–12.00)
Number of students	−0.645	0.287	5.069	0.024	0.52 (0.30–0.92)
School location	−0.484	0.203	5.657	0.017	0.62 (0.41–0.92)
Establish an infectious disease prevention and control system	0.279	0.264	1.121	0.290	1.32 (0.79–2.22)
Carry out physical examinations for freshmen	−0.853	0.266	10.242	0.001	0.43 (0.25–0.72)
Carry out morning and afternoon inspection	−0.029	0.339	0.007	0.932	0.97 (0.50–1.89)
Carry out illness absence and cause tracking registration	−3.027	0.274	122.246	0.000	0.05 (0.03–0.08)
Carry out TB prevention and control knowledge propaganda	−0.154	0.218	0.498	0.481	0.86 (0.56–1.32)
Dormitories and classrooms are regularly ventilated with open windows	−1.855	0.371	25.007	0.001	0.16 (0.08–0.32)
Strictly implement the management of sick student's suspension from school	−1.053	0.447	5.540	0.019	0.35 (0.15–0.84)
Seek timely medical consultation	−1.126	0.372	9.134	0.003	0.32 (0.16–0.67)
Set up a school infirmary	−0.212	0.417	0.259	0.611	0.81 (0.36–1.83)
Set up full-time and part-time school doctors	0.052	0.435	0.014	0.904	1.05 (0.45–2.47)
Campus health supervision	−0.077	0.221	0.123	0.726	0.93 (0.60–1.43)
Constant	0.102	0.365	0.078	0.780	

**Table 4:** Multivariate logistic regression analysis of tuberculosis aggregation in schools in Wuhan (*n* = 994).

**Independent variable and constant**	**β-value**	**Standard deviation**	**Wald *χ^2^***	***p*-value**	**OR (95% CI)**
School boarding	2.028	0.208	95.338	0.001	7.60 (5.51–11.42)
Number of students	−0.691	0.278	6.167	0.013	0.50 (0.29–0.86)
School location	−0.506	0.201	6.367	0.012	0.60 (0.41–0.89)
Carry out physical examinations for freshmen	−0.812	0.262	9.596	0.002	0.44 (0.27–0.74)
Carry out illness absence and cause tracking registration	−3.029	0.269	126.718	0.001	0.05 (0.03–0.08)
Dormitories and classrooms are regularly ventilated with windows	−1.864	0.366	25.918	0.001	0.16 (0.08–0.32)
Strictly implement the management of sick student's suspension from school	−1.029	0.437	5.551	0.018	0.36 (0.15–0.84)
Seek timely medical consultation	−1.154	0.371	9.672	0.002	0.32 (0.15–0.65)
Constant	0.332	0.295	1.268	0.260	

## Discussion

Between 2017 and 2022, the overall epidemic situation of school tuberculosis in Wuhan gradually declined from 20.75/100,000 in 2017 to 9.91/100,000 in 2022. However, the epidemic situation of tuberculosis aggregation is rising, which is similar to the rising trend of the epidemic situation of tuberculosis aggregation in schools nationwide (6). The main reason may be the promulgation of the “Standards for the Prevention and Control of Tuberculosis in Schools (2017)” by the National Health and Family Planning Commission in 2017 (10) and the “Notice on Strengthening the Physical Examination of College Students in Hubei Province” by the Hubei Provincial Department of Education and the Price Bureau (11). In addition, Wuhan Municipal Health Bureau purchased Pure Protein Derivative of Tuberculin (TB–PPD) and free preventive and therapeutic intervention drugs with municipal financial matching funds so that all kinds of schools, especially colleges and universities, entered the school for physical examination. Tuberculosis epidemic screening and strictly implement the management of sick students' suspension from school were successfully and strictly implemented, achieving the goal of “freshmen's internal defense input, illness leaving school for control” and gradually weaving and strengthening the tuberculosis epidemic prevention and control network.

This study shows that the cluster epidemic of tuberculosis mainly occurs in universities and high schools, with universities accounting for nearly 60% of Wuhan's student population, mainly concentrated in Hongshan District, Donghu High-tech Zone District, Jiangxia District, and Qiaokou District; middle school students are mainly concentrated in Wuchang District, Xinzhou District, and Huangpi District. The proportion of tuberculosis cluster epidemic in schools near urban areas was similar to that in far urban areas (**χ^2^** = 2.379, *P* = 0.123). The proportion of cluster epidemics in universities was significantly higher than in middle schools (**χ^2^** = 105.160, *P* = 0.000). There was a difference in the change in the trend of the proportion of cluster epidemics between universities and middle schools over the past 6 years. During the management and control period of COVID-19, students' teaching was carried out online through the family indoor network teaching and video clock in learning. Junior and senior high school students were under great learning pressure, especially those who were about to graduate. They lacked physical exercise and needed parents' supervision and education. They often stayed together for a long time. These factors may have increased the probability of infection in the family. In addition, COVID-19′s management and control also increased the delay of patients' medical treatment as in other places and the difficulty of centralized screening organized by the school (12–14), leading to the delay of disease control departments in handling the epidemic. This delay in disease identification and control could have helped the occurrence and spread of tuberculosis cluster epidemics in the school.

The infection rate among boys in the cluster epidemic is significantly higher than that of girls (**χ^2^** = 12.970, *P* = 0.000), which may be related to boys' poor hygiene habits, irregular work and rest, and more opportunities for extensive social contact (15, 16). In middle school, the second and third years of high school were prone to the cluster epidemic. In college, the second and third years were mainly at risk. The patients were mainly students from high epidemic areas. The main reasons may be as follows: First, some schools did not seriously implement physical examinations and routine physical for freshmen. Second, students' academic pressure, bad living habits, mild symptoms, and late medical treatment led to cluster onset (17, 18).

The incidence of symptoms among students in the cluster epidemic in Wuhan is only 36.69%, and the clinical symptoms of tuberculosis among students with positive pathogens are significantly higher than those with negative pathogens, which is related to the number and virulence of pathogenic bacteria. The more pathogenic the bacteria are, the more harmful they are to the lungs and the more obvious the symptoms are. Laboratory sputum tests showed that the pathogen-positive rate of students was 26.26% (73/278), among which the resistance rate of rifampicin or isoniazide was 6.47% (18/278). There was no significant difference in the resistance rate of rifampicin or isoniazide between high school students and university students and the entire population of pulmonary tuberculosis patients in Wuhan for 6 years. The resistance rate of rifampicin or isoniazide in a cluster epidemic of tuberculosis in schools (18/278) was higher than that of all students registered locally in Wuhan in 2017–2022, which was 3.22% (59/1,830); there was a significant difference between them (**χ^2^** = 7.247, *p* = 0.007) and it was also higher than pulmonary tuberculosis patients in the entire population of Wuhan, which was 2.89% (975/33,682). There was a significant difference between them (**χ^2^** = 13.389, *p* = 0.001), which may be that the spread of drug-resistant tuberculosis occurred in two schools in the cluster epidemic, leading to increased rifampicin or isoniazid resistance rate. We also found that the symptoms of pulmonary tuberculosis among students in suburb district were mild; the positive rate of pathogens was low, and it was difficult to detect and diagnose compared to the general population, with strong concealment. It is necessary to strengthen students' active physical examination screening and molecular biological detection technology, which is consistent with the fact that students' tuberculosis patients are easily found in Wuhan every year during graduation, admission, and annual physical examination and similar to many research results (19–23).

We further analyzed the influencing factors of the cluster epidemic by multivariate logistic regression. We found that the factors school boarding, number of students in school, school location, carry out physical examination for freshmen, carry out illness absence and cause tracking registration, open windows for ventilation regularly, strictly implement the management of sick student's suspension from school, and seeking medical attention in time, had OR values of 7.600, 0.501, 0.603, 0.444, 0.048, 0.155, 0.357, and 0.315, respectively. Among them, the risk of cluster epidemic was 3.175 times and 20.833 times higher when patients failed to see a doctor in time, and the school failed to follow up on the cause, respectively.

First, these results suggest that the physical examination standard (formulated in 2010) for primary and secondary schools in Wuhan is too low at present. However, the price of latent infection reagents for tuberculosis has risen sharply, and there is no basis for charging documents from the government administrative department, which leads to insufficient funds for tuberculosis screening physical examination. Therefore, schools should strive for policies and funds to strictly implement the daily TB prevention and control measures, especially the physical examination for freshmen, ventilation, and daily disinfection in public places such as dormitories and classrooms. Schools with high epidemic situations should conduct physical examinations every year and include TB screening items (PPD test and chest x-ray DR examination).

Second, schools should strengthen active discovery. They should carry out TB prevention and control knowledge propaganda in schools in a targeted and classified manner. They should also improve teachers' and students' awareness of TB prevention and control and guide teachers and students with suspected TB symptoms to see a doctor in time. Third, schools should track the causes of students who are absent due to illness, understand the illness situation according to the medical records of students, and interact with the local disease prevention and control departments in a timely manner. Fourth, schools should strictly implement the management of sick student's dropping out of school for. They should actively assist the disease prevention and control department in standardizing the handling of the school tuberculosis epidemic, screen out sick students, especially for schools in rural areas and private schools in urban areas, and seamlessly cooperate with the health department to effectively manage sick students.

Our study had some limitations. First, the tuberculosis epidemic in the schools only counted the students registered in the Chinese infectious disease management information system who now live in Wuhan; we did not include students enrolled who were not registered in Wuhan and who had to leave school to be quarantined at home. Hence, our statistics may underestimate the actual school tuberculosis epidemic in Wuhan. Second, as no tuberculosis clusters have been found in primary schools in Wuhan to date, only universities, high schools, and junior high schools were included in this study; the findings may be inadequate when applied to primary schools or kindergartens. Third, the work of tuberculosis prevention and control in schools is a joint responsibility that needs the leadership of the government, the cooperation of the administrative departments, and the close coordination of the schools and the various centers for disease control. This study only analyzed the school-led need for implementation and improvement, so the findings may be somewhat one-sided. Despite these limitations, this study provides new insights to improve the understanding of risk factors contributing to tuberculosis aggregation in schools in an area with a large number of students. Such studies can help schools quickly identify their own weaknesses in TB prevention and control, which are essential for formulating appropriate strategies to reduce the epidemic of tuberculosis in schools.

## Conclusion

The epidemic situation of tuberculosis in schools in Wuhan is still serious. Boarding schools, especially their large population of teachers and students, lack of supervision on campus environmental health, inadequate implementation of daily tuberculosis prevention and control measures, and delayed diagnosis of sick teachers and students, increase the risk of the occurrence and spread of tuberculosis epidemic in schools. Educational institutions should be more proactive in fulfilling their regulatory responsibilities and implementing daily prevention and control measures.

## Data availability statement

The original contributions presented in the study are included in the article/supplementary material, further inquiries can be directed to the corresponding authors.

## Ethics statement

The studies involving humans were approved by ID: Ethics Committee of Wuhan Pulmonary Hospital (2022) No. 58. The studies were conducted in accordance with the local legislation and institutional requirements. Written informed consent for participation in this study was provided by the participants' legal guardians/next of kin.

## Author contributions

ZZ: Conceptualization, Funding acquisition, Writing – original draft. GWu: Data curation, Investigation, Validation, Writing – review & editing. AY: Data curation, Investigation, Validation, Writing – review & editing. JH: Formal analysis, Investigation, Writing – review & editing. WZ: Data curation, Investigation, Writing – review & editing. ZL: Data curation, Investigation, Writing – review & editing. JWu: Investigation, Writing – review & editing. LW: Formal analysis, Investigation, Writing – review & editing. XW: Data curation, Investigation, Writing – review & editing. JWa: Data curation, Supervision, Writing – review & editing. GWa: Formal analysis, Investigation, Writing – review & editing. YL: Writing – review & editing. MZ: Conceptualization, Supervision, Writing – review & editing.

## References

[1] Ma M-J Yang Y Wang H-B Zhu Y-F Fang L-Q An X-P. Transmissibility of tuberculosis among school contacts: an outbreak investigation in a boarding middle school, China. Infect Genet Evol. (2015) 32:148–55. 10.1016/j.meegid.2015.03.001 25757905 PMC4427569

[2] Gentili D Bardin A Ros E Piovesan C Ramigni M Dalmanzio M. Impact of communication measures implemented during a school tuberculosis outbreak on risk perception among parents and school staff, Italy, 2019. Int J Environ Res Public Health. (2020) 17:911. 10.3390/ijerph17030911 32024183 PMC7037209

[3] Ding SH Wang QL Pang EC. Epidemiological investigation on a cluster epidemic of tuberculosis in a school. Chin J Sch Health. (2019) 40:1423–5. 10.16835/j.cnki.1000-9817.2019.09.042

[4] Wuhan Bureau of Statistics. Wuhan Investigation Team of National Bureau of statistics. Wuhan Statistical Yearbook. China Statistics Press (2020). p. 228–9.Available online at: https://tjj.wuhan.gov.cn/tjfw/tjnj/202312/t20231204_2312633.shtml (accessed May 25, 2024).

[5] Legal Department of the National Health and Family Planning Commission. Notice on Issuing Two Compulsory Health Industry Standards, such as Classification of Tuberculosis. [EB/OL ]. (2017). Available online at: http://www.nhc.gov.cn/fzs/s7852d/201711/0819ad84540b4d97a1644bbc6ec4306d.shtml (accessed May 25, 2024).

[6] General General Office of National Health Commission General General Office of Ministry of Education. About printing and Distributing “guidelines for tuberculosis prevention and control in Chinese schools” [EB/OL ]. (2020). Available online at: http://www.gov.cn/zhengce/zhengceku/2020-12/05/Content_5567137.htm (accessed May 25, 2024).

[7] Zhang ZB Wang GY Wang XJ Lu ZQ Ren X. Delay in treatment and influencing factors of student tuberculosis patients in Wuhan from 2011 to 2018. Chin J Sch Health. (2020) 41:1368–71. 10.16835/j.cnki.1000-9817.2020.09.024

[8] Zhao XX Yang P Gai R Mei L Wang X Xu L. Determinants of health care-seeking delay among tuberculosis patients in Shandong Province, China. Eur J Public Health. (2014) 757–61. 10.1093/eurpub/ckt113 23975893

[9] Li WH Feng HY Zhong MH Dai YJ Yan Li Zhong XG. Delay on care-seeking and influencing factors among adolescent tuberculosis patients in Dongguan City from 2009 to 2018. Chin J Sch Health. (2021) 42:265–9. 10.16835/j.cnki.1000-9817.2021.02.026

[10] General General Office of National Health and Family Planning Commission General General Office of Ministry of Education. About printing and Distributing “school tuberculosis prevention and control work specification (2017 Edition)”[EB/OL]. (2017). Available online at: http://www.moe.gov.cn/srcsite/A17/moe_943/s3285/201707/t20170727_310182.html (accessed May 25, 2024).

[11] Hubei Hubei Provincial Department of Education Provincial Health and Family Planning Commission Provincial Price Bureau. Printing and Distributing the Notice on Strengthening the Physical Examination of College Students[EB/OL]. (2018). Available online at: http://www.hubei.gov.cn/zwgk/bmdt/201808/t20180822_1332197.shtml (accessed May 25, 2024).

[12] Di Gennaro F Gualano G Timelli L Vittozzi P Di Bari V Libertone R. Increase in tuberculosis diagnostic delay during first wave of the COVID-19 pandemic: data from an Italian Infectious Disease Referral Hospital. Antibiotics. (2021) 10:272. 10.3390/antibiotics10030272 33800406 PMC7998965

[13] Han E Nabity SA Dasgupta-Tsinikas S Guevara RE Moore M Kadakia A. Tuberculosis diagnostic delays and treatment outcomes among patients with COVID-19, California, USA, 2020. Emerg Infect Dis. (2024) 30:136–40. 10.3201/eid3001.230924 38147063 PMC10756354

[14] Feyisa JW Kitila KM Lemu JC Hunde MD Hunde AD. Healthcare-seeking delay during COVID-19 pandemic among tuberculosis patients in Ilubabor zone health facilities, south-west Ethiopia. SAGE Open Med. (2022) 10:20503121221142469. 10.1177/20503121221142469 36532950 PMC9749068

[15] Wang XJ Fu Q Zhang ZB Lu ZQ Tian D Nan J. Delay on care-seeking and related influencing factors among tuberculosis patients in Wuhan, 2008-2017. Chin J Epidemiol. (2019) 40:643–7. 10.3760/cma.j.issn.0254-6450.2019.06.008 31238612

[16] Zhang XL Jiang J Fu Y Li Y Wang FX Zhang L. Screening of contacts with tuberculosis in schools in Suzhou, Jiangsu Province analysis. Chin T medicine. (2020) 20:139–41. 10.13604/j.cnki.46-1064/r.2020.02.10

[17] Gong HZ Han C Yang FL Wang CF Wang JL Wang MS. Treatment delay in childhood pleural tuberculosis and associated factors. BMC Infect Dis. (2020) 20:7590447. 10.1186/s12879-020-05496-4 33109109 PMC7590447

[18] Fang Y Ma Y Lu Q Sun J Pei Y. An outbreak of pulmonary tuberculosis and a follow-up investigation of latent tuberculosis in a high school in an eastern city in China, 2016-2019. PLoS ONE. (2021) 16:e0247564. 10.1371/journal.pone.0247564 33626108 PMC7904191

[19] Liu Q L Li Z Wang YL Chang C. Analysis on Influencing Factors of tuberculosis treatment intention of middle school students by health belief model. Chin J Dis Cont. (2019) 23:60–4. 10.16462/j.cnki.zhjbkz.2019.01.013

[20] Zhang ZB Wang TP Xie H Tian D Nan J Lu ZQ. Analysis on the characteristics of tuberculosis among students in Wuhan city from 2010 to 2014. Chin J Sch Health. (2016) 37:1908–10. 10.16835/j.cnki.1000-9817.2016.12.048

[21] Li H Liu C Liang M Liu D Zhao B Shi J. Tuberculosis outbreak in an educational institution in Henan Province, China. Front Public Health. (2021) 9:737488. 10.3389/fpubh.2021.737488 34712640 PMC8545879

[22] Gao FH Li Y Bian WJ Zhang F. Epidemiological characteristics and diagnosis and treatment management of tuberculosis among students in Zibo City from 2008 to 2017. China J Sch Health. (2018) 39:1891–4. 10.16835/j.cnki.1000-9817.2018.12.038

[23] Zhang Y Zhan B Hao X Wang W Zhang X Fang C. Factors associated with diagnostic delay of pulmonary tuberculosis among children and adolescents in Quzhou, China: results from the surveillance data 2011-2021. BMC Infect Dis. (2023) 23:541. 10.1186/s12879-023-08516-1 37596514 PMC10439644

